# Migraine and menopause as converging factors in brain vulnerability: a hypothesis-driven perspective

**DOI:** 10.1186/s10194-026-02404-0

**Published:** 2026-06-04

**Authors:** Parisa Gazerani

**Affiliations:** 1 Department of Life Science and Health, Faculty of Health Sciences, Oslo Metropolitan University, Oslo, Norway; 2 ADvanced hEalth Intelligence and Brain-insPired Technologies (ADEPT), Faculty of Technology, Art and Design (TKD), Oslo Metropolitan University, Oslo, Norway

**Keywords:** Migraine, Menopause, Brain aging, Neurodegeneration, Women’s brain health, Estrogen decline, Neuroinflammation, Mitochondrial dysfunction, Central sensitization, White matter hyperintensities, Cognitive decline, Sex differences, Neuroendocrine transition

## Abstract

Migraine is a highly prevalent neurological disorder with a pronounced female predominance, yet its relationship with brain aging remains incompletely understood. Across the female lifespan, migraine interacts with neuroendocrine transitions, particularly menopause, a period characterized by substantial hormonal, metabolic, and neuroimmune changes. This hypothesis-driven narrative perspective synthesizes evidence across clinical, neuroimaging, and mechanistic domains to explore whether migraine, particularly in chronic or hormonally sensitive forms, may reflect altered neural vulnerability in a subset of women across midlife. A conceptual neuroendocrine framework is proposed in which recurrent migraine-related processes, including network stress, neuroinflammatory signaling, and bioenergetic demand, and altered sensory processing, may interact with estrogen-dependent regulatory mechanisms across adulthood. The menopausal transition, characterized by hormonal instability followed by sustained estrogen decline, may represent a period during which previously compensated neural differences become more clinically apparent. Evidence from neuroimaging and biomarker studies suggests partial overlap between migraine-related alterations and biological processes implicated in brain aging; however, findings remain heterogeneous and are not consistently linked to long-term cognitive outcomes. Within this framework, migraine is not interpreted as a deterministic or causal factor in neurodegeneration, but rather as a potential clinical indicator of context-dependent alterations in neural vulnerability influenced by comorbidities, hormonal factors, and individual resilience. The proposed model is exploratory and highlights the need for longitudinal and mechanistic studies to clarify whether migraine-related features have relevance for long-term brain health. By integrating perspectives from neuroscience, endocrinology, and pain research, this work aims to generate testable hypotheses and to support further development of sex-informed approaches to studying brain aging in women.

## Introduction

Migraine has traditionally been conceptualized as a recurrent neurological disorder. However, advances in neuroimaging and systems neuroscience have expanded this view. Evidence suggests that migraine is associated with alterations in brain networks involved in sensory processing, vascular function, metabolism, and neuroimmune signaling, extending beyond transient attack-related dysfunction [1–4]. These observations raise the possibility that migraine may reflect not only recurrent disturbance but also broader differences in neural function or regulation over time, although this interpretation remains to be clarified.

This question becomes particularly relevant in women, who experience migraine at substantially higher rates than men across the lifespan [5, 6]. Women also undergo major neuroendocrine transitions that influence brain physiology, among which the menopausal transition represents a significant biological reorganization affecting neuronal energetics, synaptic regulation, vascular signaling, and inflammatory balance [7–9]. At the same time, midlife is increasingly recognized as a period during which trajectories of cognitive aging and neurodegenerative risk begin to emerge [10, 11]. Despite these overlapping timelines, migraine, menopause, and brain aging have largely been investigated as separate domains.

This narrative, hypothesis-driven perspective brings these fields into a unified conceptual framework by exploring whether migraine, particularly in hormonally sensitive women, may be associated with variability in brain aging trajectories. By positioning migraine within a lifespan and sex-specific neuroscience perspective, this framework aims to examine how hormonal transitions, chronic pain biology, and neural resilience may interact to shape women’s brain health across aging, while acknowledging the current limitations of the evidence base.

### Scope and aim

Migraine is one of the most prevalent and disabling neurological disorders worldwide and exhibits a marked female predominance [5, 12]. This sex disparity is most pronounced during the reproductive years and is strongly linked to fluctuations in estrogen and other sex hormones [6, 13]. Despite the substantial individual and societal burden of migraine, particularly in recurrent and chronic forms, it has been relatively underexplored within the broader context of long-term neurological function or brain aging. Emerging evidence suggests that chronic migraine may be associated with structural brain alterations, cognitive symptoms, and neuroinflammatory activity [14–17], although the clinical significance and long-term implications of these findings remain under active investigation.

This narrative, hypothesis-driven perspective provides a conceptual synthesis of potential convergence between migraine, menopause, and neurodegenerative risk in women. Specifically, it explores whether the menopausal transition, an inflection point characterized by neuroendocrine and metabolic reorganization, may be associated with the emergence or increased visibility of underlying neural vulnerability in women with a history of migraine [7–9]. Drawing on epidemiological, mechanistic, neuroimaging, and cognitive research, the review integrates evidence on partially overlapping biological pathways that may be relevant to brain aging processes, while recognizing that these associations are heterogeneous and not yet fully established.

The aim is to consolidate current knowledge while also advancing a hypothesis-driven framework intended to guide future research on sex-specific aspects of brain aging in women with migraine. This work does not seek to establish causal relationships or validated predictive markers but rather to identify potential areas of convergence that warrant further investigation. Importantly, menopause does not affect all individuals with migraine uniformly. While hormonal stabilization may alleviate migraine in some individuals, others experience persistent or evolving symptoms frequently accompanied by cognitive complaints often described as brain fog [6, 13, 18]. This heterogeneity highlights the importance of considering menopause not solely as a reproductive milestone but as a context in which pre-existing neurological differences may become more apparent.

### Relevance to women’s brain health and aging

Women’s brain health remains comparatively underrepresented in neurology and aging research despite well-established sex-specific differences in neurobiology, disease trajectories, and treatment responses [19, 20]. The menopausal transition represents a critical phase in the female lifespan, historically framed as a reproductive milestone but increasingly recognized as a systemic and neurological transition point [7–9]. Declining estrogen levels have been associated with changes in synaptic plasticity, neurovascular coupling, mitochondrial bioenergetics, and neuroimmune regulation, processes that are also implicated in cognitive aging [21–23].

During this period, many women experience changes in migraine patterns alongside cognitive symptoms commonly described as brain fog, including impaired attention, reduced processing efficiency, and memory lapses [6, 13]. These manifestations are often addressed as independent clinical entities; however, their temporal co-occurrence may reflect broader changes in brain function and regulatory processes during midlife. From a lifespan neuroscience perspective, menopause may therefore represent a window during which pre-existing neurological differences or susceptibilities become more apparent, although the mechanisms underlying these observations remain incompletely understood.

Hormone therapy (HT), widely used to manage menopausal symptoms, introduces additional complexity into this relationship. Observational and clinical studies report heterogeneous effects on migraine and cognition depending on formulation, timing, and individual risk profile [24–26]. In particular, evidence suggests that the timing of initiation relative to menopause onset may influence neurological outcomes, consistent with the proposed “critical window” hypothesis. Consequently, HT may function as both a biological modifier and a potential confounding factor within the migraine–menopause–brain aging interface.

### Overview of hypothesis and conceptual framework

This hypothesis-driven narrative review synthesizes emerging clinical, mechanistic, and neuroimaging evidence to outline a conceptual framework. It explores the possibility that migraine, particularly in chronic or hormonally modulated forms, may be associated with differences in neural resilience or vulnerability across midlife in women. Biological overlap between migraine and processes relevant to neurodegeneration has been described at the level of neuroinflammatory signaling, mitochondrial function, and large-scale network organization [1, 2, 15, 21, 27], although these mechanisms are not specific and are shared across multiple neurological and systemic conditions.

Within this framework, migraine is reconsidered not solely as a recurrent neurological disorder but as a potential clinical characteristic that may reflect variability in neural resilience within a broader, sex-specific context of brain aging. Situating migraine within hormonal and neurobiological transitions of midlife allows for the outline of a multidimensional and testable model of women’s brain health. Although requiring longitudinal validation, this framework is intended to generate hypotheses and to inform future research rather than to support clinical risk identification or prediction at this stage.

**Fig. 1:**
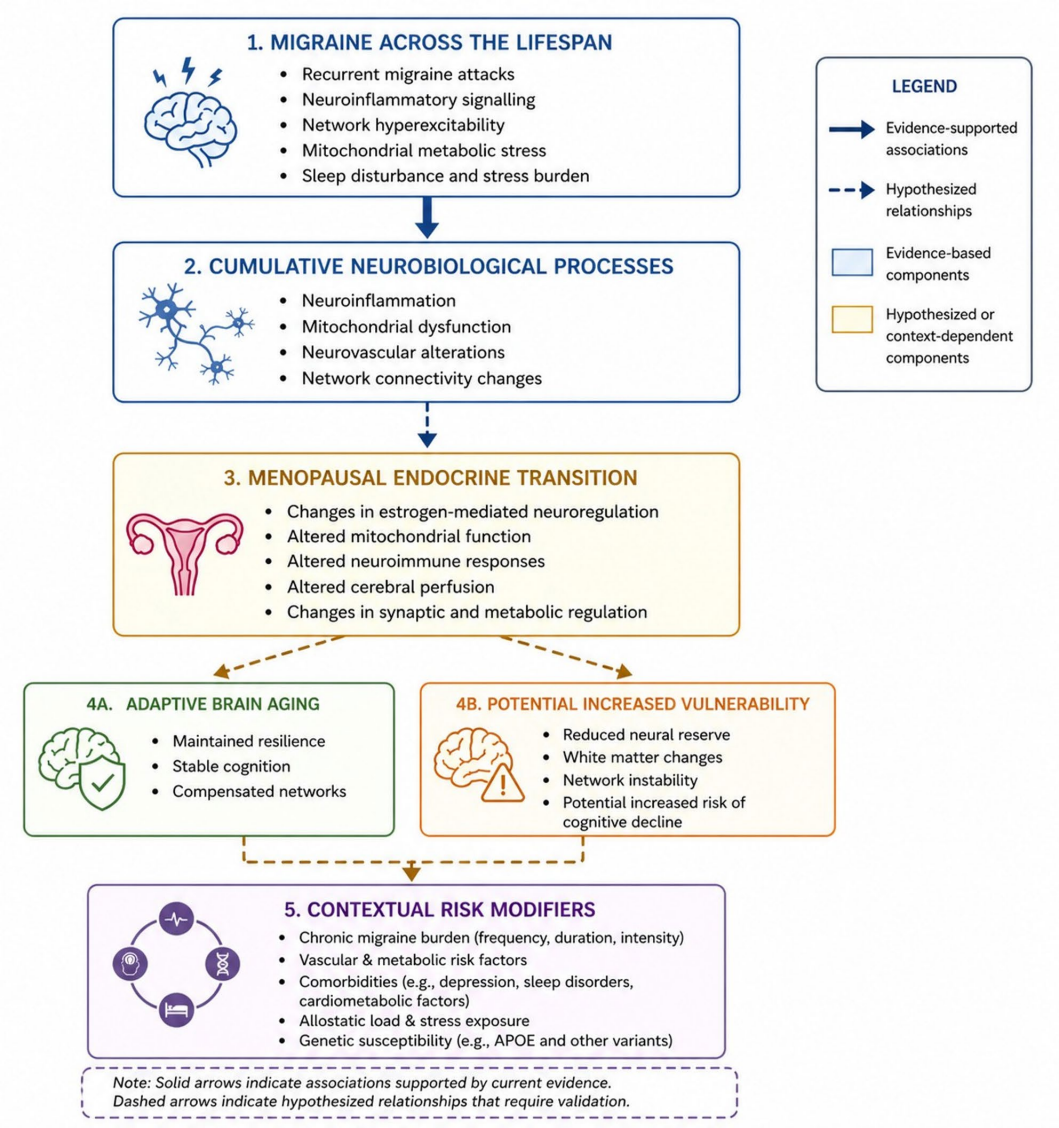
Conceptual framework illustrating proposed relationships between migraine, the menopausal transition, and brain aging in women. Migraine across the female lifespan is associated with recurring biological processes, including neuroinflammatory signaling, mitochondrial dysfunction, neurovascular alterations, and large-scale network changes. During the menopausal transition, hormonal instability followed by sustained estrogen decline is associated with reduced regulatory buffering of these systems, which may increase the visibility of pre-existing neural differences in some individuals. Solid arrows represent associations supported by existing empirical evidence, whereas dashed arrows indicate hypothesized or unvalidated relationships. The model is conceptual and hypothesis-driven and does not imply causality or predictive validity. Note: The figure has been developed by the author based on an original conceptual sketch and refined using AI-assisted digital illustration tools

The conceptual framework proposed here is illustrated in Fig. 1, which summarizes how migraine may interact with menopausal endocrine transition and cumulative physiological stress in ways that may be relevant to brain aging and cognitive function.

Migraine is therefore conceptualized not as a direct cause of neurodegeneration but as a potential context-dependent clinical characteristic that may reflect underlying variability in neural resilience during midlife. Importantly, the proposed relationships are based on associative and mechanistic evidence and should be interpreted as exploratory, requiring further validation in longitudinal and population-based studies.

## Migraine across the female lifespan

### Epidemiology and female predominance

Migraine is a highly prevalent neurological disorder affecting more than one billion individuals worldwide and is characterized by a pronounced female predominance that evolves across the lifespan [5, 28]. Prior to puberty, migraine prevalence is similar in boys and girls; however, following menarche, the prevalence increases markedly in females, supporting an association with sex hormone dynamics and migraine susceptibility [6, 13, 29, 30]. By adolescence, girls are approximately twice as likely as boys to report migraine, and this disparity persists throughout the reproductive years [5, 31].

Women between 20 and 50 years of age carry the greatest migraine burden. During this period, attacks are often more frequent, longer lasting, and more disabling compared with those observed in men [32–34]. Hormone-sensitive migraine patterns, including menstrual-related migraine, are common and particularly associated with phases of declining estrogen levels, such as the late luteal phase preceding menstruation [35, 36]. Women are also more likely to experience migraine with aura, a subtype associated with elevated vascular risk [37, 38], and are disproportionately affected by chronic migraine, defined as ≥ 15 headache days per month for more than three months, with at least eight days fulfilling migraine criteria according to the International Classification of Headache Disorders (ICHD-3) [39].

As women approach midlife, endocrine regulation becomes increasingly unstable in the years leading up to menopause. The menopausal transition unfolds gradually and encompasses biologically distinct phases characterized by fluctuating and ultimately declining estrogen levels [7, 40]. Variability in hormonal exposure across the lifespan, including pregnancy, lactation-associated hypoestrogenism, and surgical menopause, further contributes to heterogeneity in migraine expression and progression [36, 41]. A summary of hormonal stages and their typical associations with migraine patterns across the lifespan is presented in Table 1.

**Table 1:** Hormonal phases across the female lifespan and typical associations with migraine expression

Hormonal Phase	Estrogen Pattern	Typical Migraine Association	Notes
Childhood	Low, relatively stable	Low prevalence	Minimal hormonal influence
Menarche	Rising and cyclic	Increased onset incidence	Frequently represents the first clinical manifestation
Reproductive years	Cyclic fluctuations	Variable frequency and severity	Menstrual-related migraine common
Pregnancy	Sustained high and stable	Often improvement or remission of migraine attacks	Improvement is most pronounced during the second and third trimesters
Lactation	Suppressed (hypoestrogenic)	Variable response	Prolactin-mediated estrogen suppression; interindividual variability
Perimenopause	Markedly fluctuating, declining	Often worsening or changes in migraine frequency, severity, or phenotype	Hormonal instability increases attack susceptibility
Surgical menopause	Abrupt estrogen withdrawal	Acute worsening in some individuals	Bilateral oophorectomy produces a sudden endocrine transition
Natural menopause	Gradual cessation	Variable course, including improvement, persistence, or change in attack characteristics	Some individuals develop a new aura or altered triggers
Postmenopause	Persistently low and stable	Partial improvement in many individuals, with persistence or altered triggers in others	Non-hormonal triggers are increasingly relevant

Note: Associations shown in the table reflect general patterns reported in epidemiological, clinical, and review studies on migraine across hormonal life stages. They are intended as a conceptual summary and do not imply uniform responses or causal relationships across individuals. Supporting references include [6, 13, 35, 36, 40, 41]

Following perimenopause, menopause is defined retrospectively after twelve consecutive months of amenorrhea, during which estrogen levels stabilize at persistently low concentrations [40]. Migraine patterns frequently shift during this period, although the direction and magnitude of change vary across individuals. Many women with hormonally sensitive migraine experience improvement after hormonal stabilization, whereas others continue to experience attacks with altered triggers and clinical characteristics [42, 43]. In the postmenopausal phase, non-hormonal contributors, including sleep disturbance, psychosocial stress, and metabolic dysregulation, may assume greater importance [44]. Some women also report cognitive symptoms such as slowed processing or subjective brain fog; however, these symptoms are generally mild, often transient, and not consistently linked to objective cognitive decline [43–46].

Abrupt estrogen withdrawal, as occurs in surgical menopause, may precipitate acute worsening of migraine in some individuals, particularly in the absence of hormone therapy [41, 47]. Conversely, lactation represents another hypoestrogenic state in which migraine patterns vary considerably between individuals, reflecting differences in hormonal sensitivity and neuroendocrine adaptation [36].

Although epidemiological data suggest that the sex gap in migraine prevalence narrows after menopause, findings remain heterogeneous across cohorts [5, 42]. Approximately one-third of women continue to experience migraine beyond menopause, indicating that hormonal mechanisms alone do not fully explain migraine persistence or chronification [43]. These lifespan patterns highlight migraine as a condition closely intertwined with female neuroendocrine development and provide essential context for exploring its potential relevance to long-term brain health.

### Migraine subtypes and chronification

Migraine is a heterogeneous neurological disorder comprising multiple clinical subtypes that differ in symptom expression, vascular risk, hormonal sensitivity, and potentially long-term brain outcomes. The current diagnostic classification, based on ICHD-3, remains predominantly symptom-based and lacks routinely used objective biomarkers, despite advances in neuroimaging, neurophysiology, and molecular research. This limitation may obscure biologically meaningful differences in underlying mechanisms and potentially in long-term neurological outcomes [39, 48–50].

The two principal clinical phenotypes are migraine without aura (MO) and migraine with aura (MA). Migraine without aura is characterized by recurrent unilateral pulsatile headaches lasting 4–72 h and typically accompanied by nausea, photophobia, phonophobia, and worsening with routine physical activity. Migraine with aura involves transient focal neurological symptoms, most commonly visual, but also sensory, language, or motor disturbances that precede or accompany the headache phase and are typically gradual in onset and fully reversible [39].

Migraine with aura is of particular interest in relation to long-term brain health. Epidemiological studies report associations with ischemic stroke, silent cerebral infarction, and vascular comorbidity, particularly in women exposed to additional vascular risk factors such as smoking or estrogen-containing contraceptives [37, 38, 51–54]. However, these associations are influenced by co-existing vascular risk factors and do not imply a direct causal relationship.

Beyond episodic migraine, chronic migraine represents a major clinical transition. Chronification reflects not only increased attack frequency but is also associated with alterations in central pain processing, including central sensitization, impaired descending inhibitory control, and markers suggestive of low-grade neuroinflammatory activity. Psychiatric comorbidity, particularly depression and anxiety, is also common [2, 55–57].

Women are disproportionately affected by chronic migraine, particularly during hormonally dynamic life stages, including adolescence, the postpartum period, and the menopausal transition [58]. This pattern likely reflects interactions between endocrine modulation of trigeminovascular excitability and broader psychosocial and systemic factors. Chronic migraine may therefore be considered a multidimensional brain state shaped by biological, hormonal, and environmental influences rather than solely a frequency-defined disorder [6, 29, 30, 36].

Despite refinement of clinical criteria, migraine subtypes remain insufficiently biologically validated. Neuroimaging studies have identified recurring features, including white matter hyperintensities, altered thalamocortical connectivity, and brainstem hyperresponsiveness, but these findings have limited predictive value at the individual level. Likewise, fluid-based biomarkers capable of distinguishing migraine subtypes remain limited in clinical utility [59–61].

Emerging multimodal approaches integrating neuroimaging, endocrine profiling, inflammatory markers, and digital phenotyping are being explored as potential avenues for biologically informed stratification. Such approaches may be particularly relevant for studying midlife women, where migraine evolution coincides with neuroendocrine transitions that have been associated with processes related to brain aging and cognitive changes [62–65].

Understanding how migraine phenotypes evolve across the female lifespan and interact with reproductive milestones and aging processes [66] remains an important area for further research within precision neurology and sex-informed clinical care [67].

### Endocrine modulation of migraine: Beyond estrogen?

Estrogen plays a central role in modulating migraine susceptibility across the female lifespan. Importantly, migraine risk appears to depend less on absolute estrogen levels than on the rate and direction of hormonal change, with rapid estrogen withdrawal lowering the threshold for migraine attack initiation [6, 68, 69]. This dynamic regulation is most evident during hormonally unstable periods such as menstruation, postpartum, and the menopausal transition.

Menstrual migraine is closely associated with perimenstrual estrogen decline, which has been associated with increased cortical excitability and facilitation of cortical spreading depolarization (CSD), a key neurophysiological mechanism implicated in migraine aura and trigeminovascular activation [61, 70–72]. Conversely, pregnancy, particularly during the second and third trimesters, is characterized by sustained high and stable estrogen concentrations and is frequently associated with improvement or remission of migraine [73, 74]. In contrast, the postpartum and perimenopausal periods, marked by hormonal volatility and abrupt withdrawal, are often associated with migraine worsening or phenotypic change [75, 76].

Although estrogen has dominated migraine research [43, 77], accumulating evidence suggests that migraine expression is influenced by a broader endocrine network integrating stress, circadian, metabolic, and neurotrophic signaling pathways [49, 78–80]. In addition to estrogen, other reproductive hormones—including progesterone and gonadotropins (follicle-stimulating hormone [FSH] and luteinizing hormone [LH]) also undergo substantial changes across the menopausal transition and may influence both migraine expression and brain function, although these pathways remain less well characterized. These systems may become particularly relevant in midlife, when endocrine aging intersects with increasing neuroinflammatory and metabolic changes associated with aging.

Dysregulation of the hypothalamic–pituitary–adrenal (HPA) axis represents one such pathway. Chronic stress exposure and altered cortisol rhythms have been associated with heightened pain sensitivity, impaired descending pain modulation, and altered inflammatory signaling in migraine [49, 81, 82]. Sustained cortisol elevation may also be associated with hippocampal vulnerability and cognitive inefficiency, suggesting a potential link between stress biology, migraine chronification, and processes related to brain aging [57, 83–85].

Circadian regulation represents another important modulatory system. Melatonin has been reported to be reduced in some individuals with migraine, exerts antioxidant and anti-inflammatory effects and contributes to sleep–wake stability [86, 87]. Disrupted melatonergic signaling may therefore contribute to migraine susceptibility and age-related neurobiological stress, while clinical studies suggest potential therapeutic benefit in selected populations [88].

Metabolic and endocrine factors further extend this framework. Thyroid dysfunction, particularly autoimmune hypothyroidism, has been reported to be more prevalent among women with migraine and may contribute to fatigue, mood disturbance, and cognitive slowing [89, 90]. Similarly, insulin resistance and metabolic dysregulation have been associated with cortical hyperexcitability, oxidative stress, and vascular dysfunction [91, 92], mechanisms that are also implicated in neurodegenerative processes. Declines in growth hormone (GH) and insulin-like growth factor-1 (IGF-1), both important for synaptic plasticity and neuronal repair, may be associated with reduced neural resilience during aging [93].

Collectively, these observations suggest that migraine may be considered not solely a hormonally triggered disorder but also a condition embedded within a broader neuroendocrine regulatory system. Interactions among stress physiology, sleep regulation, metabolic health, and neurotrophic signaling may shape migraine trajectories across the lifespan and are also relevant to long-term brain health. These relationships are summarized in Table 2.

**Table 2:** Non–sex hormone endocrine systems linking migraine pathophysiology and brain aging

Hormone/System	Mechanistic Role in Migraine	Relevance to Brain Aging and Cognitive Decline
Cortisol (HPA axis)	Stress reactivity, impaired descending pain modulation, and promotion of neuroinflammation	Hippocampal vulnerability, impaired synaptic plasticity, and memory decline
Melatonin	Regulation of sleep–wake cycles, anti-inflammatory and antioxidant effects; reduced levels reported in migraine	Circadian disruption, impaired glymphatic clearance, and increased oxidative stress
Thyroid hormones (T3/T4)	Modulation of cerebral metabolism, vascular tone, and mood regulation	Hypothyroidism is associated with cognitive slowing and reduced metabolic efficiency
Insulin / Metabolic signaling	Cortical excitability, endothelial dysfunction, systemic inflammation	Insulin resistance linked to Alzheimer’s disease risk and vascular cognitive impairment
GH/IGF-1 axis	Support of synaptic plasticity, neuronal repair, and neurogenesis	Age-related decline is associated with reduced brain repair capacity and structural atrophy

Abbreviations: HPA, hypothalamic–pituitary–adrenal axis; T3, triiodothyronine; T4, thyroxine; GH, growth hormone; IGF-1, insulin-like growth factor-1

Note: Associations reflect general patterns derived from experimental, clinical, and review studies on neuroendocrine regulation in migraine and brain aging and do not imply causal relationships or uniform effects across individuals. Supporting references include [49, 57, 81–93]

## Menopause and neurological changes: a critical transition in female brain aging

### The neuroendocrine and neurological shift of menopause

Menopause represents a major neuroendocrine transition across the female lifespan, characterized by cessation of ovarian function and a sustained decline in circulating estrogens, along with broader changes in other reproductive hormones. Menopause is increasingly recognized as a neuroendocrine and systemic inflection point with important implications for brain metabolism, network stability, and processes related to cognitive aging [7, 9, 94, 95].

Estrogen exerts broad neuromodulatory and potentially neuroprotective effects throughout the central nervous system, particularly within regions involved in cognition, affect regulation, and pain processing, including the hippocampus, prefrontal cortex, and insular cortex [23, 96–98]. At the cellular level, estrogen supports synaptic plasticity, enhances mitochondrial bioenergetics, modulates major neurotransmitter systems, and suppresses pro-inflammatory signaling pathways [21, 22, 99–101]. These actions are thought to contribute to neural efficiency and adaptive resilience during the reproductive years.

Experimental and translational studies further indicate that estrogen modulates cortical excitability, thalamocortical integration, and trigeminovascular signaling, processes relevant to migraine susceptibility and chronification [6, 29, 30, 36, 102]. Hormonal fluctuation, rather than absolute estrogen deficiency alone, appears particularly relevant in migraine biology, as rapid hormonal shifts may increase susceptibility to cortical spreading depolarization, the neurophysiological substrate underlying migraine aura [61, 103]. During the menopausal transition, this regulatory environment undergoes progressive reorganization. Declining estrogen availability is associated with alterations in synaptic homeostasis, mitochondrial function, and neuroimmune balance, which may contribute to reduced metabolic flexibility and diminished compensatory capacity [104–107]. In this context, previously compensated neural vulnerabilities may become more clinically apparent, particularly in individuals with hormonally sensitive or chronic migraine.

Neuroimaging studies in perimenopausal and postmenopausal women provide convergent observations consistent with these changes. Reported findings include changes in cerebral glucose metabolism, region-specific gray matter alterations in hippocampal and prefrontal regions, and changes in white matter integrity within networks supporting executive and sensory integration [8, 108–110]. Some longitudinal and observational studies suggest that hormone therapy initiated near menopause onset may be associated with partial attenuation of selected metabolic or structural changes, consistent with the concept of a potential window of neuroplastic responsiveness during endocrine transition [111–113].

Importantly, menopause represents a dynamic process rather than a discrete event. The perimenopausal phase is characterized by pronounced hormonal variability, including irregular estrogen fluctuations and intermittent withdrawal phases. This instability is commonly associated with vasomotor symptoms, sleep disturbance, mood variability, and cognitive complaints, often described as brain fog [114–116]. In women with pre-existing migraine, such fluctuations may coincide with changes in attack frequency or phenotype, reflecting heightened network sensitivity during endocrine transition.

At the systems level, reorganization of the hypothalamic–pituitary–ovarian axis also interacts with interconnected regulatory pathways, including the hypothalamic–pituitary–adrenal stress axis and circadian networks [8, 117, 118]. Altered stress responsivity, impaired descending pain modulation, and disrupted sensory processing may therefore emerge in parallel, potentially providing a biological basis for the co-occurrence of migraine symptoms and cognitive complaints during midlife.

Taken together, menopause may be understood as a multisystem neurological recalibration rather than solely a reproductive endpoint. Within the proposed neuroendocrine unmasking framework, loss of estrogenic modulation may contribute to the emergence of latent neural vulnerabilities accumulated earlier in life. For women with chronic migraine, this transition may represent a period of increased susceptibility in which prior network stress, inflammatory priming, and bioenergetic strain converge, potentially influencing trajectories related to cognitive aging [19, 20, 119]. However, substantial interindividual variability exists, and not all women experience adverse neurological or cognitive outcomes during menopause, underscoring the need for longitudinal and stratified investigations.

### Cognitive changes during menopause: mechanisms and clinical features

Cognitive complaints represent some of the most frequently reported non-reproductive symptoms during the menopausal transition. Commonly described as brain fog, these experiences include reduced attention, word-finding difficulty, impaired working memory, and slowed processing speed [112, 120, 121]. Although often transient, such symptoms can affect daily functioning, particularly in midlife women who are otherwise cognitively healthy and professionally active.

Importantly, menopause does not uniformly result in objective cognitive decline. Deficits detected on standardized neuropsychological testing are typically modest [112, 122]. Nevertheless, large longitudinal cohorts, including the Study of Women’s Health Across the Nation (SWAN), demonstrate that early perimenopause is associated with modest but measurable reductions in verbal memory and attention independent of mood, sleep disturbance, and sociodemographic factors [108, 123]. In many women, cognitive performance stabilizes after menopause, although recovery varies and may depend on individual differences in neurological resilience [112, 123].

The neurobiology of menopause-associated cognitive change is multifactorial and is thought to be closely linked to the withdrawal of estrogen’s neuromodulatory influence on brain regions supporting memory and executive control. Estrogen is known to support synaptic plasticity, modulates cholinergic and glutamatergic signaling, and maintains cerebral glucose metabolism within the hippocampus, prefrontal cortex, and posterior cingulate cortex regions also implicated in migraine-related network dysfunction and in processes associated with early neurodegenerative change [124–126].

Several interacting mechanisms appear to contribute to cognitive symptoms during this transition, as outlined below.

#### Reduced cerebral perfusion

Through regulation of endothelial nitric oxide synthase and vascular signaling pathways, estrogen supports neurovascular coupling and cerebral autoregulation [23, 99]. Estrogen withdrawal has been associated with reduced regional perfusion, particularly within frontal and parietal networks involved in attention and working memory [8, 127, 128]. Arterial spin-labeling MRI studies demonstrate decreased cerebral blood flow in postmenopausal women, an effect that may be more pronounced in individuals with migraine, where endothelial dysfunction and episodic cortical hypoperfusion have been reported [105, 129].

#### Altered functional network connectivity

Estrogen is known to modulate large-scale brain networks, including the default mode, central executive, and salience networks [130, 131]. Functional MRI studies have reported reduced intra-network coherence and impaired cross-network coordination during perimenopause, correlating with subjective cognitive inefficiency [132, 133]. Similar connectivity alterations are also described in migraine, particularly within insular and salience circuitry, suggesting potentially convergent mechanisms of network instability [134, 135].

#### Oxidative stress and neuroinflammation

Estrogen contributes to mitochondrial efficiency and suppresses pro-inflammatory signaling pathways, thereby maintaining redox balance in neural tissue [136, 137]. Menopause may reduce this regulatory buffering, contributing to increased oxidative stress and low-grade neuroinflammation [136, 138]. In women with migraine, especially those with metabolic or vascular comorbidities, these processes may already be relatively primed, potentially increasing susceptibility to neuroimmune amplification and blood-brain barrier vulnerability [139, 140].

#### Sleep disruption and circadian dysregulation

Sleep disturbances are common during perimenopause and arise from vasomotor instability, hormonal fluctuation, and age-related changes in melatonin signaling [141–143]. Impaired sleep can affect memory consolidation, reduce glymphatic clearance of neurotoxic metabolites, and may increase cortical excitability [144–146]. Because sleep disturbance both triggers and perpetuates migraine, this mechanism may represent an important intersection between menopausal symptoms and headache chronification [147–149].

#### Mood disturbances and limbic–cortical dysregulation

Rates of depression and anxiety have been reported to increase during the menopausal transition, partly reflecting hormonal modulation of monoaminergic systems [150]. Mood disorders are associated with impairments in attention, processing speed, and memory encoding [151, 152]. Neuroimaging studies demonstrate altered activity within the amygdala, anterior cingulate cortex, and dorsolateral prefrontal cortex, regions implicated in both migraine and menopause-related affective symptoms, highlighting potential shared circuit-level vulnerability [1, 8, 153, 154].

Collectively, these changes describe a transient but biologically meaningful neurophysiological state characterized by reduced metabolic efficiency, altered network integration, and increased inflammatory tone [75, 155]. While many women regain cognitive stability after menopause, individuals with lower cognitive reserve or higher baseline vulnerability or pre-existing neurological burden, including chronic migraine, may exhibit incomplete compensation. In such cases, menopause may not initiate neurodegeneration but may contribute to the earlier clinical expression of latent vulnerabilities, potentially functioning as a neurological tipping point [156, 157].

Distinguishing menopause-related cognitive fluctuation from early neurodegenerative change remains challenging. Biomarker-based stratification is limited, although emerging neuroimaging and cerebrospinal fluid studies suggest partial overlap with patterns observed in preclinical Alzheimer’s disease [158, 159]. Evidence regarding hormone therapy remains heterogeneous, likely reflecting differences in timing, formulation, and population characteristics. The critical window hypothesis proposes that cognitive benefit may occur primarily when therapy is initiated near menopause onset, during preserved neural plasticity [160, 161].

For women with chronic migraine, already associated with structural brain alterations, white matter abnormalities, and changes in cognitive control, the menopausal transition may therefore represent a period of amplified vulnerability during which compensatory neural networks may be strained [41, 162]. Recognizing this interaction may be important for developing sex-specific strategies to support brain resilience and potentially mitigate long-term cognitive risk. However, considerable interindividual variability exists, and many women do not experience persistent cognitive impairment, underscoring the importance of longitudinal and individualized approaches.

### Long-term neurological vulnerabilities and neurodegenerative risk

This section examines how the menopausal transition may function as a neurological stress test, potentially amplifying pre-existing vulnerabilities in women with chronic migraine and may contribute to trajectories associated with accelerated brain aging. Neurological vulnerability can be conceptualized as a state in which subtle structural or functional alterations reduce the brain’s capacity to adapt to physiological stressors, such as endocrine transitions, inflammatory activation, or metabolic strain, without immediately producing overt disease [163, 164]. Within this framework, menopause represents a critical inflection point in brain aging, particularly among women with pre-existing vascular, cognitive, or pain-related burden [8].

#### Menopause as a potential tipping point in brain aging

During menopause, the brain undergoes recalibration in response to declining estrogenic regulation across multiple biological systems, including synaptic plasticity, neurovascular coupling, mitochondrial energetics, and neuroimmune signaling [8, 23]. While many women adapt through compensatory neuroplastic mechanisms, others may exhibit less efficient adaptation, which may result in persistent alterations resembling features associated with accelerated brain aging [165, 166].

Reported features of this potentially maladaptive trajectory include reduced cerebral perfusion within frontal and parietal cortices, altered large-scale functional connectivity, sustained low-grade neuroinflammation, mitochondrial inefficiency with increased oxidative stress, and impaired glymphatic clearance [11, 105, 145, 167]. Although not inherently pathological, these changes may lower resilience thresholds and increase susceptibility to later cognitive decline in individuals with reduced cognitive reserve, cardiometabolic risk, APOE-ε4 genotype, or chronic migraine [168–170].

#### Migraine as a risk amplifier in the aging female brain

Migraine, particularly chronic, hormonally sensitive, or aura-associated forms, shares several biological features with conditions associated with neurodegeneration. These include neuroinflammatory activation, white matter abnormalities, cortical thinning in pain-processing and associative regions, dysregulated monoaminergic and glutamatergic signaling, and instability of large-scale brain networks such as the default mode and salience systems [171, 172].

Although migraine itself is not classified as a neurodegenerative disorder, longitudinal studies suggest that individuals with longstanding migraine may exhibit structural brain alterations more frequently than non-migraine populations [1, 173, 174]. Associations with cognitive inefficiency and executive dysfunction have been reported, although causal relationships remain uncertain [175, 176].

The menopausal transition may therefore represent a period of potentially synergistic vulnerability in which cumulative migraine-related network stress intersects with reduced hormonal neuroprotection and age-related metabolic changes [66, 177].

#### Neurodegenerative conditions of particular relevance

Several neurological disorders demonstrate female predominance, sensitivity to hormonal transitions, and partial mechanistic overlap with migraine biology.

Alzheimer’s disease (AD) disproportionately affects women and has been linked to estrogen-dependent regulation of amyloid processing, tau phosphorylation, and synaptic maintenance [19, 95, 101]. Neuroimaging studies demonstrate posterior cortical hypometabolism in postmenopausal women carrying APOE-ε4, potentially influenced by metabolic and inflammatory stressors also observed in chronic migraine [178, 179].

Cerebral small vessel disease (SVD) has been reported more frequently in individuals with migraine with aura and is characterized by white matter hyperintensities, silent infarcts, and microvascular dysfunction, processes that may be intensified by estrogen withdrawal and vascular comorbidity [51, 180, 181].

Vascular and mixed dementias may arise through cumulative endothelial dysfunction and silent ischemic injury. Migraine has been associated with increased risk of subclinical infarction, while postmenopausal metabolic and hypertensive changes may further elevate vascular vulnerability [102, 182].

Parkinson’s disease (PD) shows more complex sex differences but remains relevant given estrogen’s modulatory effects on dopaminergic systems [183]. Although links between migraine and PD remain speculative, overlapping network alterations and motor circuit sensitivity warrant further investigation [184, 185].

These conditions are not caused by migraine or menopause alone but share potentially converging biological substrates, including neuroinflammation, mitochondrial dysfunction, cerebral hypoperfusion, and network reorganization [186, 187].

#### Conceptual integration: Migraine – menopause – neurodegeneration

Taken together, the available evidence supports a conceptual model in which midlife women with chronic or hormonally sensitive migraine may represent a subgroup with increased vulnerability during the menopausal transition. Within this framework, cumulative migraine-related structural and functional alterations may contribute to the priming of neural systems, while estrogen withdrawal may destabilize compensatory regulatory mechanisms and age-related metabolic and inflammatory changes may further reduce adaptive capacity.

This interaction may not immediately produce overt neurodegenerative disease but could contribute to reduced cognitive resilience and potentially contribute to accelerated progression in predisposed individuals. The converging pathways underlying this proposed interaction are summarized in Table 3.

**Table 3:** Converging biological pathways linking menopause, migraine, and neurodegenerative risk in women

Mechanistic Pathway	Menopause-Related Changes	Evidence in Migraine Biology	Linked to Neurodegenerative Risk	Examples of Relevant Conditions
Neuroinflammation	↑ Microglial reactivity, ↓ estrogen anti-inflammatory control	↑ CGRP, IL-6, TNF-α; chronic pain state	Promotes tau phosphorylation, neuronal injury	AD, SVD
Cerebral Hypoperfusion	↓ Estrogen-mediated vasodilation, impaired autoregulation	Cortical spreading depolarization (CSD), endothelial dysfunction	Brain ischemia, silent infarcts, WM lesions	VaD, SVD
Mitochondrial Dysfunction	↓ Bioenergetic efficiency, ↑ ROS	Mitochondrial stress in chronic migraine	Energy failure, oxidative damage	AD, PD
Functional Connectivity	Disruption in DMN, salience, executive networks	Network instability in chronic migraine and aura	Early network breakdown	AD
White Matter Changes	Microvascular stress, estrogen withdrawal	WM hyperintensities, silent infarcts	Associated with cognitive decline, reduced processing speed	SVD, Mixed dementia
Glymphatic Dysfunction	↓ Sleep quality, melatonin dysregulation	Sleep-related migraine triggers, impaired clearance	Impaired β-amyloid and tau clearance	AD
Mood and Cognitive Reserve	Depression, anxiety, ↓ cognitive reserve postmenopause	Higher psychiatric comorbidity in migraine	Amplifies vulnerability to cognitive decline	AD, MCI

Abbreviations: AD, Alzheimer’s disease; CGRP, calcitonin gene-related peptide; CSD, cortical spreading depolarization; DMN, default mode network; IL-6, interleukin-6; MCI, mild cognitive impairment; PD, Parkinson’s disease; ROS, reactive oxygen species; SVD, cerebral small vessel disease; TNF-α, tumor necrosis factor-α; VaD, vascular dementia; WM, white matter

Note: Associations reflect general patterns derived from experimental, clinical, and epidemiological studies on menopause, migraine biology, and neurodegenerative processes and do not imply causal relationships or uniform effects across individuals. Supporting references include [8, 11, 23, 51, 95, 102, 145, 167, 171–174, 180–182, 186, 187]

Importantly, these relationships are associative and hypothesis-driven rather than causal, and substantial interindividual variability exists. Longitudinal studies integrating biological, imaging, and clinical data are required to determine the extent to which these pathways translate into measurable neurodegenerative risk.

## Sex differences in neurodegenerative risk and brain aging outcomes

### Sex-specific epidemiological trends and cognitive trajectories in brain aging

Building on the hypothesis that migraine may amplify neurological vulnerability during hormonal transitions, this section situates these mechanisms within broader sex-specific trajectories of cognitive aging and neurodegenerative risk. Understanding how brain aging differs between women and men provides important context for interpreting migraine as a potential modifier of long-term neurological outcomes [19, 20].

Epidemiological evidence consistently demonstrates that women bear a disproportionate burden of age-related neurodegenerative disease, particularly AD. Globally, nearly two-thirds of individuals diagnosed with AD are women [188]. Although this disparity has historically been attributed to longer female life expectancy, accumulating evidence suggests that longevity alone cannot fully explain the difference, pointing instead toward a combination of sex-specific biological, hormonal, and sociocultural determinants of brain aging [104].

Large longitudinal cohorts, including the Mayo Clinic Study of Aging, the Framingham Heart Study, and the Rotterdam Study, demonstrate meaningful differences in cognitive aging trajectories between women and men [189–191]. Women often exhibit stronger performance in verbal memory and language domains during midlife and early aging, which may mask early neuropathology during clinical evaluation [192]. However, once decline becomes clinically apparent, women have been reported to show steeper trajectories of deterioration, including more pronounced hippocampal atrophy, greater functional impairment, and faster loss of independence [193, 194]. This pattern has been described as a paradox of preserved function followed by more rapid decline [195].

Biomarker investigations from cohorts such as the Alzheimer’s Disease Neuroimaging Initiative (ADNI) and the Australian Imaging, Biomarker & Lifestyle Flagship Study of Ageing (AIBL) further support the presence of sex-specific differences in vulnerability [196]. Women with comparable amyloid-β and tau burden have been reported to demonstrate greater clinical severity and faster progression than men, suggesting possible differences in resilience thresholds or compensatory capacity [197, 198]. Neuroimaging studies similarly report more pronounced temporoparietal hypometabolism and accelerated hippocampal volume loss in women during disease progression [104].

Midlife exposures may play an important modulatory role. Cardiometabolic factors, including hypertension, diabetes, and dyslipidemia, are often underrecognized or undertreated in women and have been associated with disproportionate downstream cognitive consequences [11, 199]. The menopausal transition may represent a period of increased susceptibility during which metabolic, inflammatory, and vascular stressors may exert amplified neurological effects [7, 200].

Sex differences in cognitive reserve further shape aging outcomes. Advantages in language and social cognition may delay clinical detection of impairment in women, yet once deficits emerge, functional decline may progress more rapidly [192, 201]. These patterns likely reflect the interaction of biological susceptibility with gendered social determinants of health, including caregiving roles and healthcare access [202].

Importantly, much of the available epidemiological evidence derives from Western populations, which may limit generalizability across global contexts [203]. Differences in education, reproductive health, and healthcare infrastructure may substantially influence observed trajectories. Survival bias should also be considered, as higher midlife cardiovascular mortality among men may partially obscure comparative cognitive vulnerability [204].

Notably, migraine remains largely absent from major cognitive aging cohorts despite its high prevalence among midlife women and its known sensitivity to hormonal transitions. Chronic or hormonally modulated migraine, particularly when accompanied by affective or metabolic comorbidity, may interact with sex-specific pathways involving neuroinflammation, vascular dysfunction, and reduced synaptic resilience [75, 171]. Incorporating migraine phenotyping into longitudinal brain aging research may therefore help identify previously underrecognized high-risk subgroups and inform strategies for early intervention. However, direct longitudinal evidence linking migraine to sex-specific cognitive aging trajectories remains limited, highlighting a key gap for future research.

### Disease burden in postmenopausal women

Postmenopausal women experience an increased burden of several age-related neurological disorders, most prominently AD, followed by VCID, SVD, and, to a lesser extent, PD and dementia with Lewy bodies (DLB) [205]. Although these conditions affect both sexes, differences in prevalence, progression, and clinical expression highlight the importance of considering sex-specific determinants of late-life neurological health [206, 207].

Women undergoing early or surgical menopause appear to face an increased risk, likely reflecting the impact of abrupt estrogen deprivation occurring before full adaptive compensatory mechanisms may develop. Longitudinal studies suggest that both the timing and duration of hypoestrogenic exposure may influence later cognitive outcomes [208]. Key sex-specific disease patterns and their potential intersections with migraine biology are summarized in Table 4.

**Table 4:** Sex-specific features of major neurodegenerative disorders in postmenopausal women and potential overlap with migraine biology

Disorder	Female-Predominant Features	Potential Overlap with Migraine Biology
AD	Higher prevalence; faster verbal memory decline; greater tau burden	Migraine with aura may share glutamatergic excitotoxicity and inflammatory pathways
SVD	More extensive white matter hyperintensities; greater cognitive impact from vascular risk	Migraine is linked to periventricular lesions, endothelial dysfunction, and cortical hypoperfusion
DLB	Later diagnosis in women; more psychiatric features; less Parkinsonism	Potential overlap with dopaminergic dysregulation and REM sleep disturbances
PD	Lower prevalence in women; more tremor-dominant phenotype; better early treatment response	Migraine-associated basal ganglia changes have been reported but are not conclusive

Abbreviations: AD, Alzheimer’s disease; DLB, dementia with Lewy bodies; PD, Parkinson’s disease; SVD, cerebral small vessel disease

Note: Associations reflect general patterns derived from epidemiological, clinical, and neurobiological studies on sex differences in neurodegenerative disorders and migraine and do not imply causal relationships or uniform effects across individuals. Supporting references include [19, 51, 95, 102, 171, 180–185, 205–208]

During the menopausal transition and early postmenopausal years, many women report cognitive symptoms, including attentional lapses, slowed processing, and subjective memory difficulty. While these symptoms are frequently transient, they may reflect a temporary reduction in neurocognitive efficiency rather than overt pathology [209]. In women with chronic migraine, however, overlapping mechanisms, including altered cerebral perfusion, neuroinflammatory priming, and network instability, may reduce compensatory reserve and potentially increase susceptibility to persistent cognitive difficulties [210].

Distinguishing menopause-associated cognitive fluctuation from early neurodegenerative change remains clinically challenging. Persistent or progressive symptoms may therefore warrant closer clinical evaluation and risk factor management. From a population health perspective, integrating migraine phenotyping into longitudinal studies of cognitive aging may help refine dementia risk stratification and inform sex-informed strategies to preserve brain health across the aging process.

Together, these observations provide a biological and epidemiological context for examining whether migraine may function as a marker of altered neural resilience during midlife, a possibility explored in the following section. However, direct causal links between menopause, migraine, and neurodegenerative disease remain to be established, and current evidence is largely associative.

## Migraine as a midlife marker of brain vulnerability

Within the conceptual framework proposed in this review (Fig. 1), migraine is interpreted not as a neurodegenerative disorder but as a potential marker of altered neural resilience, which may reflect either increased vulnerability or adaptive or compensatory neurobiological responses depending on individual context. Importantly, this framework also allows for the possibility that migraine may reflect adaptive or compensatory neurobiological responses in some individuals, rather than solely a marker of vulnerability. Recurrent migraine across early and mid-adulthood may expose neural systems to repeated biological stressors, including inflammation, metabolic strain, sleep disruption, and altered sensory processing [211]. Over time, these factors may influence the brain’s capacity to adapt to additional physiological and environmental challenges across the lifespan.

The menopausal transition represents a particularly relevant phase within this trajectory. As estrogen-dependent regulatory systems decline, may become more clinically apparent rather than newly generated, reflecting a shift in system-level resilience rather than the emergence of entirely new pathology. Cognitive complaints frequently reported during midlife may therefore reflect reduced adaptive capacity in susceptible individuals rather than overt neurodegenerative change.

Importantly, migraine should not be interpreted as a deterministic pathway to neurodegeneration. Many individuals with migraine maintain stable cognitive trajectories across the lifespan. Instead, migraine history may function as a longitudinal marker of vulnerability that interacts with genetic, metabolic, and environmental modifiers. Neuroimaging studies provide convergent evidence in support of this perspective. Across several cohorts, individuals with migraine, particularly those with chronic migraine or migraine with aura, demonstrate alterations in both white matter [212] and grey matter architecture [213].

White matter hyperintensities (WMHs), visible on T2-weighted or FLAIR MRI sequences, represent markers of cerebral small vessel disease and vascular brain aging [214]. Population-based studies such as the CAMERA [215] and ARIC [216] cohorts report increased WMH burden in migraine populations, particularly among women and individuals with migraine with aura [217]. Lesions frequently localize to frontal and periventricular regions and may reflect cumulative vascular instability and neuroimmune activation associated with recurrent migraine activity over time, although causal relationships remain to be established [217, 218].

Grey matter volume differences have also been reported in regions involved in sensory processing, emotional regulation, and cognitive control [213]. Meta-analyses identify structural changes in the anterior cingulate cortex, insula, somatosensory cortex, and parahippocampal regions. Functional imaging studies further demonstrate altered connectivity within default mode, salience, and frontoparietal networks, suggesting that structural differences may influence large-scale network efficiency [1].

Independent of migraine, menopause has been associated with structural alterations in estrogen-sensitive brain regions, including the hippocampus and medial prefrontal cortex [8]. The coexistence of migraine-related and endocrine-related changes therefore suggests a model of dual vulnerability in midlife women. Repeated physiological stress in chronic migraine may also impose a cumulative burden across neuroendocrine, immune, autonomic, and metabolic systems. Persistent pain, sleep disruption, affective comorbidity, and hormonal instability together contribute to increased allostatic load [219].

Physiological alterations observed in chronic migraine, including altered cortisol dynamics, inflammatory activation, reduced autonomic regulation, and bioenergetic inefficiency, parallel biological processes associated with accelerated aging. Midlife endocrine transition may further amplify this imbalance by reducing hormonal buffering of stress physiology and neuroimmune signaling. Within this integrative framework, chronic migraine may be understood as a potential sentinel marker of cumulative neurobiological strain, becoming particularly relevant during midlife brain aging.

## Clinical and research implications for women’s brain health

Reconceptualizing migraine within a brain-aging framework carries important implications for clinical practice and neuroscience research. Rather than viewing migraine solely as a recurrent pain disorder, this perspective suggests that chronic and hormonally sensitive migraine may may serve as a clinically accessible indicator of altered neural resilience during midlife.

Clinical evaluation of women with migraine may therefore benefit from extending beyond headache frequency to include cognitive symptoms, sleep quality, mood status, menopausal stage, and cardiometabolic health. Integrating neurological and hormonal assessment may facilitate earlier identification of individuals with increased vulnerability in cognitive aging trajectories.

Advances in neuroimaging, inflammatory biology, and endocrine research also suggest the potential for biologically informed risk stratification. Although current markers are not suitable as standalone diagnostic tools, combining structural imaging, systemic inflammatory indicators, and hormonal context may help identify potentially modifiable risk trajectories during periods of relative neuroplastic preservation.

Digital health technologies can also offer additional opportunities for longitudinal monitoring. Wearable sensors, mobile applications, and remote cognitive assessments can capture real-world patterns of sleep, stress physiology, and symptom fluctuation. Integration of these data streams with clinical and biomarker information may support more individualized monitoring and early intervention strategies.

Ultimately, integrating migraine biology with broader frameworks of women’s brain health may contribute to shifting clinical practice from reactive symptom control toward more proactive approaches aimed at preserving neural resilience across midlife and aging.

## Limitations and future directions

This review has several limitations that should be acknowledged. First, it is a narrative and hypothesis-driven synthesis rather than a systematic review and may therefore be subject to selection bias in the literature included.

Second, much of the evidence linking migraine, menopause, and neurodegenerative risk remains largely associative rather than causal, and longitudinal studies directly examining these interactions are still limited.

Third, migraine is a heterogeneous condition, and variability in subtype classification, hormonal sensitivity, and disease course may influence reported associations may influence reported associations across studies.

Fourth, the proposed framework emphasizes vulnerability pathways, while resilience mechanisms, including cognitive reserve, adaptive neuroplasticity, and protective lifestyle factors, remain comparatively less well characterized.

Finally, most available data derive from Western populations, limiting generalizability across diverse settings.

Future research should prioritize longitudinal, multimodal approaches integrating neuroimaging, endocrine profiling, and digital phenotyping to better clarify potential causal pathways and identify both vulnerability and resilience factors across the female lifespan.

## Conclusion

This review examined whether migraine, particularly chronic and hormonally sensitive migraine in midlife women, may represent a marker of increased brain vulnerability of increased brain vulnerability emerging at the intersection of endocrine transition, neuroinflammation, and cumulative physiological stress. Evidence from neuroscience, endocrinology, and pain research suggests that migraine, menopause, and neurodegenerative risk intersect through partially shared biological pathways.

Within the proposed neuroendocrine unmasking framework, the menopausal transition may function as a critical inflection point in female brain aging. Declining estrogenic regulation affects neuroimmune balance, mitochondrial metabolism, and vascular stability, systems also implicated in migraine biology. In individuals with longstanding migraine, this transition may therefore may reveal latent neural vulnerabilities shaped by prior network stress and systemic strain.

Migraine should not be interpreted as a deterministic precursor to neurodegeneration. Most individuals with migraine maintain stable cognitive trajectories throughout life. Instead, may function as a sentinel marker, an early clinical indicator of altered neural resilience that highlights opportunities for prevention while adaptive capacity remains intact.

Recognizing migraine within a lifespan framework of women’s brain health may enable earlier identification of vulnerability and support strategies aimed at maintaining cognitive resilience across aging. At the same time, individual trajectories remain highly heterogeneous, and future research should clarify the balance between vulnerability and resilience mechanisms in women’s brain aging.

## Data Availability

No datasets were generated or analysed during the current study.
